# Memory of Infections: An Emerging Role for Natural Killer Cells

**DOI:** 10.1371/journal.ppat.1003548

**Published:** 2013-09-26

**Authors:** Alexander Rölle, Julia Pollmann, Adelheid Cerwenka

**Affiliations:** Innate Immunity Group, German Cancer Research Center (DKFZ), Heidelberg, Germany; Columbia University, United States of America

Many previous reports on Natural Killer (NK) cells highlighted their ability to form the proverbial first line of defense against a variety of viral infections and malignancies. NK cells have been considered a part of innate immunity, characterized by invariant, germ-line encoded receptors for the recognition of pathogens and infected cells. In contrast, somatic rearrangement of receptor genes, the clonal expansion of antigen-specific cells, and the ability to mount a more potent memory response upon secondary challenge are traditionally considered hallmarks of T and B cells belonging to the adaptive immune system.

More recently, exciting new data are challenging this conventional view [1]. A growing body of evidence indicates that under certain experimental conditions, NK cells share some of the features of adaptive immune cells. For instance, in mice infected with murine cytomegalovirus (MCMV), an NK cell subset expands in an antigen-dependent manner reminiscent of the clonal expansion of adaptive immune cells [2]. This NK cell expansion is associated with long-lasting functional changes similar to features of memory T cell populations. Resemblances between NK and T cells are not only limited to their response kinetics and certain functions, but also comprise characteristics of homeostatic proliferation, development, and differentiation [3].

Currently, a clear consensus on how the term “memory” is defined in NK cell biology is lacking. Throughout this article we will refer to memory NK cells, if these NK cells respond more potently to a second challenge with the same antigen they had initially encountered (Figure 1). The term “memory-like” NK cells will be used when long-lasting functional alterations are induced, e.g. by cytokines without clear evidence of antigen involvement (Figure 1).

**Figure 1:**
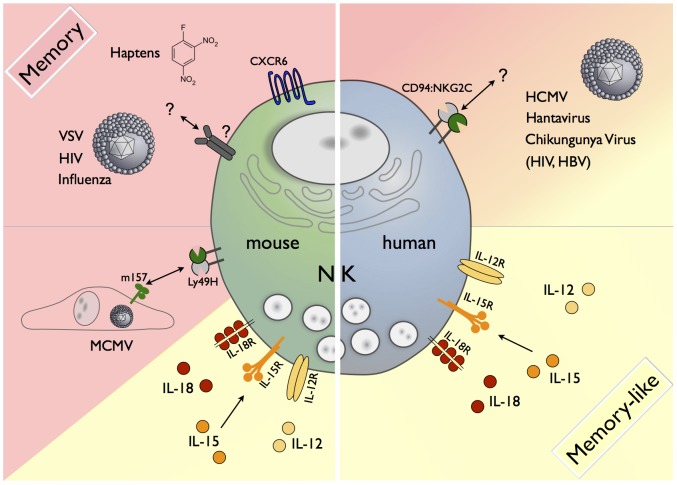
Memory and memory-like NK cells in mice and humans. A variety of factors contribute to the generation of memory or memory-like cells. In the mouse, CXCR6^+^ NK cells from the liver can mediate antigen-specific memory responses against haptens and viral antigens of VSV, HIV, and influenza via a yet-unknown receptor(s). During MCMV infection, the viral m157 protein is recognized by a subset of NK cells carrying the activating Ly49H receptor, resulting in the formation of m157-specific Ly49H^+^ memory NK cells. Memory-like NK cells in mice and humans can be generated by short-term stimulation with IL-12/15/18. A subset of human NK cells expressing the activating CD94/NKG2C receptor expands in response to the as-yet undefined antigens in HCMV, Hantavirus, Chikungunya Virus, HIV, and HBV infection.

## Features of Memory NK Cells in Mice

The first evidence of NK cell-mediated recall responses was obtained in a model of hapten-induced contact hypersensitivity in *Rag2^−/−^* mice deficient in adaptive immune cells [4]. In these mice different haptens—compounds that chemically modify proteins—provoked a hypersensitivity reaction that was transferrable to naive animals by adoptive NK cell transfer. The transferred NK cells reacted only against the same hapten that they had encountered during initial sensitization, and not against structurally related haptens. Notably, the NK cells mediating this response were confined to the liver and expressed the chemokine receptor CXCR6. A follow-up report from the same group extended this concept to NK cell responses against several viruses, namely to influenza, vesicular stomatitis virus (VSV), and human immunodeficiency virus (HIV) [5]. NK cells from animals vaccinated with viral antigens protected naive mice against a lethal challenge with the sensitizing virus. The ability to mount a recall response persisted for several months. In both studies, the required receptor-ligand interactions and signaling pathways leading to the generation of antigen-specific memory NK cells remained elusive.

In murine cytomegalovirus (MCMV) infection binding of the activating NK cell receptor Ly49H to the viral protein m157 that is expressed on the surface of infected cells is a prerequisite for the proliferation of Ly49H^+^ NK cells. More recently, a study demonstrated that, after clearance of the infection, these cells decreased in number, but a persisting population was detectable for several months [2]. These NK cells were functionally more competent than naive NK cells and 10 times more efficient in mediating protection against MCMV challenge in adoptive transfer experiments.

## NK Cell Subset Expansion in Response to Infections in Humans

In humans, clear evidence for NK cell memory is lacking. In analogy to results obtained in the MCMV mouse model, expansion of certain human NK cell subsets was observed in various viral infections, which might reflect a first step for the subsequent generation of memory NK cells.

A first report suggested that infection with human cytomegalovirus (HCMV) skews the NK cell receptor repertoire toward the activating CD94/NKG2C receptor that is usually expressed on less than 10% of total NK cells in peripheral blood [6]. Later studies corroborated this finding *in vitro* [7] and demonstrated a similar expansion of NKG2C+ NK cells (to up to 70% of all NK cells) in recipients of solid organ, allogeneic cell, or umbilical cord blood transplantation during episodes of primary HCMV infection or reactivation [8]–[10]. After expansion, the NKG2C+ NK cells were more potent producers of IFN-γ than their NKG2C-counterparts and expressed CD57, a marker of terminal differentiation [9]. A follow-up study demonstrated that after hematopoietic cell transplantation, NKG2C+ NK cells from CMV-positive donors expanded in CMV+ recipients, whereas NKG2C+ NK cells from CMV-negative donors did not, suggesting the existence of a secondary response against CMV antigen [11].

Other viral infections were also reported to have an impact on the NKG2C+ NK cell subset. During acute Hantavirus infection, NKG2C+ NK cells expanded three- to four-fold compared to uninfected controls and declined only slowly over the course of several months [12]. In Chikungunya virus infection, a similar expansion was described and the percentage of NKG2C+ NK cells inversely correlated with viral titers [13]. An increased frequency of NKG2C+ cells was also linked to other infections such as HIV [14] and Hepatitis B Virus (HBV) [15]. Of note, in both cases this increase was only observed in patients who were HCMV-seropositive. Accordingly, a cohort carrying a gene deletion in the *KLRC2* gene, encoding the NKG2C protein, had an increased risk of contracting HIV, a more rapid disease progression, and higher viral titers prior to initiation of treatment [16]. These data suggest that the NKG2C receptor is actively participating in the immune response against the virus, rather than representing a mere marker of a responding subset. Of note, HLA-E, a non-classical MHC class I molecule presenting peptides from MHC class I-derived leader sequences, serves as a ligand for CD94/NKG2C and is known to be upregulated in HCMV [17], Hantavirus [12], and HIV infection [18].

Four of the studies discussed above describe a predominance of the inhibitory NK cell receptor KIR2DL2/3 receptor within the NKG2C+ NK cells [8], [12], [13], [15]. The complex interactions between the highly polymorphic gene families of inhibitory NK cell receptors of the Killer-cell Immunoglobulin-like Receptor (KIR) family and their MHC class I ligands have been a topic of intense research. Their stochastic expression gives rise to diverse repertoires of NK cells. Thus, it is possible that subset expansion is linked to the presence or absence of certain KIRs in a particular MHC class I environment.

## A Role for Soluble Factors in the Generation of Memory and Memory-Like NK Cells

In addition to specific antigens that drive the expansion of NK subsets and the formation of NK cell memory in mice, the involvement of cytokines for the generation of long-lived NK cell populations with superior effector function attracts increasing attention. The proliferation of MCMV-specific Ly49H^+^ NK cells was shown to be dependent on the IL-12R and the downstream transcription factor STAT4 [19]. Recent studies with both mouse and human NK cells indicate that a short *in vitro* exposure to a combination of IL-12, IL-15, and IL-18 (IL-12/15/18) yields memory-like NK cells that display superior effector function and longevity *in vitro* and *in vivo*, and those properties were also inheritable to daughter cells [20]–[22]. Exposure of NK cells to IL-12/15/18 upregulated the IL-2Rα chain (CD25) [22]–[23], making these cells more responsive to IL-2. Indeed, IL-2-producing CD4+ T cells contributed to the maintenance of the reactivated NK cell populations *in vivo* [22]. It remains to be tested whether cytokines produced during different infectious diseases drive the formation of memory-like NK cells that contribute to protection against a re-challenge.

## The Molecular Basis for NK Cell Memory: Open Questions

A key task in this emerging field is the identification of receptors and factors that initiate and shape the responses against haptens or influenza, VSV, or HIV, ultimately leading to the generation and maintenance of memory NK cells. It will be crucial to extend this work to model systems in which adaptive immunity is present to assess the relative importance of NK cells in the protection against re-challenge by infectious agents. The definition of markers of NK cell memory or memory-like function would facilitate these studies dramatically. The intriguing liver-restriction of NK cells mediating memory responses against haptens and certain viruses will certainly be another focus of future investigations.

In humans, most reports suggesting the existence of NK cell memory highlight the increased proportion of NKG2C+ NK cells in viral infections. The broad range of viruses that was reported to trigger the expansion of this subset might indicate that this phenomenon relies on an induced or altered self-ligand rather than on a shared pathogen-derived structure. Provided that NKG2C is functionally involved in the antiviral immune responses, in some infections the upregulation of the ligand, HLA-E, would provide a straightforward explanation for the expansion of NK cells carrying its cognate receptor NKG2C. Nonetheless, evidence for a causal role of HLA-E in NK cell subset expansion during an ongoing infection is lacking. It awaits further investigation as to whether NKG2C and/or other activating receptors participate in processes leading to NK memory in a diverse range of infections.

Progress in our understanding of how NK cells can exert functions resembling adaptive immune responses might have implications beyond conceptually questioning the classic division of the immune system into an innate and an adaptive branch and our general view of immunological memory. Novel insights on memory NK cells could also have a strong impact on the design of next generation vaccines against a variety of pathogens.
